# Regulation of the Interferon Response by lncRNAs in HCV Infection

**DOI:** 10.3389/fmicb.2018.00181

**Published:** 2018-02-16

**Authors:** Saba Valadkhan, Puri Fortes

**Affiliations:** ^1^Department of Molecular Biology and Microbiology, Case Western Reserve University School of Medicine, Cleveland, OH, United States; ^2^Center for Applied Medical Research, Department of Gene Therapy and Hepatology, Navarra Institute for Health Research (IdiSNA), University of Navarra, Pamplona, Spain

**Keywords:** type I IFN, lncRNAs, HCV, proviral, antiviral, IFN response

## Abstract

The interferon (IFN) response is a critical component of the innate immunity antiviral pathways in mammalians. IFN signaling results in increased expression of cellular factors that block key steps in the viral replication cycle. Many IFN-induced antiviral factors act through decreasing viral entry, replication, transcription, translation, packaging and release. However, these effects are also deleterious for the viability of the cell, which necessitates a tight control over the magnitude and duration of the IFN response. This is partially achieved through the IFN-mediated activation of negative regulatory factors that help in termination of the IFN response and return to a normal homeostatic state. Such built-in negative regulatory mechanisms are frequently hijacked by viruses such as the Hepatitis C virus (HCV) to increase viral replication and productive infections. We and others have shown that long non-coding RNAs (lncRNAs) play prominent roles in regulation of the IFN response. Activation of the IFN cascade alters the expression of a large number of lncRNAs, many of which are directly induced by the JAK/STAT pathway and thus, resemble the well-studied protein-coding interferon-stimulated genes (ISGs). While only a handful of IFN- and virally induced lncRNAs have been characterized, recent studies have identified several lncRNAs that act as positive or negative regulators of expression of ISGs during the IFN response. A number of such regulatory lncRNAs have multiple ISG targets, while others act on a single neighboring ISG. Another group of studied lncRNAs act further upstream and regulate the expression of IFN genes or factors that sense the presence of viral genome or replication products. The large number of unstudied IFN- and virally induced lncRNAs makes it highly likely that future studies will reveal a much greater share for this class of transcripts in regulation of the antiviral response. In addition to their physiological roles, the expression of such lncRNAs is frequently modulated by virally encoded factors to interfere with the antiviral response and promote viral replication, thus making them ideal targets for therapeutic intervention.

## Long Non-Coding RNAs as a New Class of Regulatory Molecules

High throughput transcriptome studies in the last decade have led to the discovery of 1000s of novel RNA molecules that do not appear to code for functional peptides. These transcripts, named the long non-coding RNAs (lncRNAs), can be found in both eukaryotes and prokaryotes. However, they are especially abundant in more complex eukaryotes including animals and plants (Rinn and Chang, 2012; Morris and Mattick, 2014). Interestingly, their abundance seems to correlate with the level of complexity of the organism, for example while protein-coding sequences constitute < 2% of the human genome, a significantly larger fraction of our genomes is coding for lncRNAs (ENCODE Project Consortium, 2012; Clark et al., 2013). lncRNAs have diverse modes of biogenesis and mechanism of action and constitute a heterogeneous group of transcripts. Many of them are transcribed by RNA polymerase II and are spliced and polyadenylated, although on average they contain fewer introns compared to protein-coding genes (Carninci et al., 2005; Derrien et al., 2012; Niazi and Valadkhan, 2012). However, several studied lncRNAs are RNA polymerase I and III transcripts and a significant fraction of lncRNAs are not polyadenylated (Carninci et al., 2005; Derrien et al., 2012; Djebali et al., 2012). Similarly, they show a wide heterogeneity in size and can range from a couple of hundred to tens of thousands of nucleotides in length. An arbitrary lower length limit of 200 nucleotides which has been proposed for this class of RNAs should not be applied too strictly, as it mainly serves as a general guideline for distinguishing lncRNAs from the small non-coding RNA such as small nuclear and nucleolar RNAs, microRNAs, etc. (Clark and Mattick, 2011; Rinn and Chang, 2012; Mattick and Rinn, 2015).

Despite intense research in recent years, the sheer number and diversity of lncRNAs have limited our understanding of the scope of function of lncRNAs in higher eukaryotes. Evidence emerging from existing research indicate that the expression of many lncRNAs is strongly dependent on the cell type and cellular state and is tightly controlled by various cellular signals (Rinn and Chang, 2012; Amaral et al., 2013). Thus, it is likely that many lncRNAs remain to be described and annotated as more cellular states are probed with high throughput transcriptomic studies. Even among the annotated lncRNAs, the vast majority remain unstudied. Finally, many alternatively processed unstudied isoforms of protein-coding RNAs do not have a significant protein-coding capacity and thus, can potentially affect the cellular function as non-coding RNAs (Carninci et al., 2005; Djebali et al., 2012; ENCODE Project Consortium, 2012). Therefore, it is likely that future research will elucidate a much larger share for lncRNAs in cellular function than currently assumed.

Nonetheless, data from the small number of lncRNAs currently studied indicate their involvement in diverse aspects of cellular function (Wapinski and Chang, 2011; Moran et al., 2012; Rinn and Chang, 2012; Amaral et al., 2013; Ulitsky and Bartel, 2013; Yang et al., 2014). Emerging data suggest that a major functional mechanism of lncRNAs involves regulation of nuclear events, including transcriptional regulation and control of the epigenetic state of chromatin (Rinn and Chang, 2012; Amaral et al., 2013; Rinn, 2014). Accordingly, localization of a lncRNA to the nuclear compartment and association with chromatin modifying complexes or transcription factors serve as potential clues into the function of a lncRNA.

**FIGURE 1:**
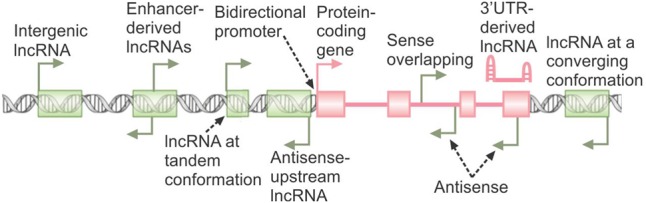
The genomic origins of lncRNAs. The broken arrows indicate the location of transcription start sites and direction of transcription. The protein-coding gene is shown in pink, with green rectangles marking the loci of non-overlapping lncRNA genes. A 3′ UTR-derived lncRNA originating from stabilization of degradation products of the protein-coding RNA is shown.

Another potential indicator of the function of a lncRNA is the genomic locus from which it originates (**Figure 1**). Genes coding for lncRNAs frequently overlap protein-coding genes or other lncRNA genes in the sense or antisense orientation with the lncRNA initiating from a different promoter. In many cases, such promoters are located within or near the 3′ UTR of protein-coding genes (Carninci et al., 2005; Derrien et al., 2012; Djebali et al., 2012). Further, many lncRNAs result from selective stabilization of a certain region of a protein-coding gene, such as the 3′ UTR (Mercer et al., 2011). Such overlapping genes may affect the biogenesis and/or function of the other genes in the locus via several potential mechanisms, ranging from epigenetic regulation of the activity of the locus, to transcriptional interference, to masking or competing with functionally critical elements of the other transcripts arising from the overlapped locus through base-pairing (Valadkhan and Nilsen, 2010). Over 10% of human genes originate from the so-called bidirectional promoters, and in many cases at least one of the two promoter-sharing genes is a lncRNA (**Figure 1**) (Adachi and Lieber, 2002; Wakano et al., 2012; Uesaka et al., 2014). Data from studied examples has shown that in such loci, one member of the pair can regulate the expression of the other transcript which originates from the same promoter (Wei et al., 2011; Uesaka et al., 2014). Another subclass of lncRNAs, those originating from promoters in enhancer loci, are thought to be crucial for the function of the enhancers from which they are transcribed (**Figure 1**) (Lam et al., 2014). Finally, many lncRNAs are located in vicinity of other genes without overlapping them or sharing architectural elements with them (**Figure 1**). Such intergenic vicinal lncRNAs can potentially affect the expression of their nearby genes via transcriptional interference or epigenetic regulation (Valadkhan and Nilsen, 2010; Rinn and Chang, 2012; Mattick and Rinn, 2015). It should, however, be mentioned that many functional lncRNAs are located far away from their target genes and thus, locus proximity or even overlap is not necessarily a requirement for a regulatory relationship. On the other hand, some lncRNAs may function in cis to regulate the expression of genes located far in the genome but in the same nuclear territory (Hacisuleyman et al., 2014).

As mentioned above, current evidence point to the involvement of lncRNAs in virtually every aspect of cellular function. Several pioneering studies in recent years have provided evidence for the critical role of lncRNAs in regulation of diverse aspects of the immune response, including both innate and adaptive immunity (Fitzgerald and Caffrey, 2014; Heward and Lindsay, 2014; Imamura and Akimitsu, 2014; Stachurska et al., 2014; Marques-Rocha et al., 2015; Satpathy and Chang, 2015; Sigdel et al., 2015; Yu A.D. et al., 2015; Yu Q. et al., 2015). While the main focus of this review is on the host-derived lncRNAs involved in the interferon arm of the antiviral response, recent research has revealed that a number of virally coded lncRNAs also affect the IFN response (summarized in Valadkhan and Gunawardane, 2015). The following sections include a discussion of the host-derived lncRNAs induced by and/or affecting the interferon response, followed by a more focused overview of the role of lncRNAs in hepatitis C infection as a well-studied example of a viral infection. Comprehensive reviews on the role of lncRNAs in development and function of the immune response are included elsewhere (Valadkhan and Gunawardane, 2015; Carpenter, 2016; Atianand et al., 2017).

## The Interferon Arm of the Antiviral Response

**FIGURE 2:**
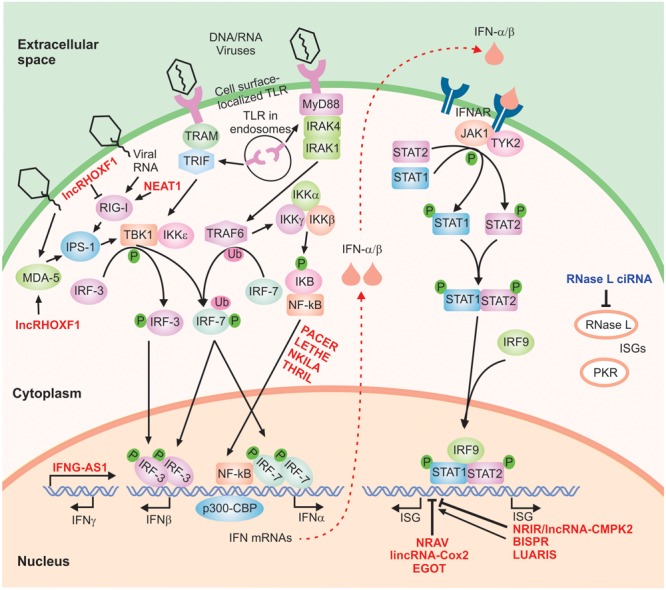
The IFN signaling cascade. A highly simplified scheme of the type I IFN signaling cascade is shown. The host-encoded regulatory lncRNAs are shown in red font. Phosphorylation and ubiquitination events are indicated by small green circles and pink ovals, respectively.

A crucial and nearly ubiquitous component of the innate immune response against viruses and many other microbial pathogens is mediated through the interferon (IFN) signaling cascade. IFNs are traditionally divided into three major groups (I, II, and III). While binding distinct receptors, type I (IFN-α, -β, -κ, -𝜀, and -ω) and type II (IFN-γ) IFNs show extensive overlap in their downstream signaling cascades and regulated genes (Hertzog et al., 2011; Pollard et al., 2013; Rusinova et al., 2013; Bolen et al., 2014). Similarly, type III IFNs (IFN-λ1, IFN-λ2, IFN-λ3, also called IL-29, IFN-λ4, IL-28A, and IL-28B, respectively), which are predominantly expressed in plasmacytoid dendritic cells in addition to a number of other cell types, bind a distinct receptor but show downstream overlap with type I IFNs (Meyer, 2009; Pollard et al., 2013; Bolen et al., 2014). The IFN response is initiated through the induction of expression of IFNs via activation of a class of cellular factors that act as the initial sensors of pathogen-associated molecular patterns. Among them, RNA sensors such as retinoic acid inducible gene I (RIG-I), melanoma differentiation-associated gene-5 (MDA5) and membrane-bound Toll-like receptors (TLR 3, 7, or 8) are particularly relevant to antiviral response against RNA viruses, although cells also contain several DNA sensors (**Figure 2**) (Hertzog and Williams, 2013; Ivashkiv and Donlin, 2014; Schneider et al., 2014). Activation of the sensor molecules, in turn, leads to signal transduction cascades and ultimately induction of the expression of genes with specific inhibitory functions against micro-organisms.

In the case of the type I IFN response, recognition of pathogen-associated patterns by RIG-I and MDA5 leads to activation of the IFN-β promoter stimulator 1 (IPS-1), while TLR3 and TLR7/TLR8 induce signaling via TRIF and MyD88, respectively (Hertzog and Williams, 2013; Ivashkiv and Donlin, 2014; Schneider et al., 2014). The activation of these pathways, in turn, will result in phosphorylation and dimerization of transcription factors including the interferon regulatory factor 3 (IRF-3), and IRF-7 followed by their nuclear translocation (**Figure 2**). Next, together with NF-κB and ATF-2/c-jun, they nucleate the formation of active transcriptional complexes through interactions with several transcription factors and regulatory proteins such as the transcriptional cofactor (CREB)-binding protein (CBP)/p300 (Kawai and Akira, 2006; Hiscott, 2007; Randall and Goodbourn, 2008; Takeuchi and Akira, 2009; Onomoto et al., 2010; Jensen and Thomsen, 2012). This, in turn, results in induction of the expression of antiviral cytokines including type I IFNs (IFN-α and IFN-β). While IFN-β is produced in most cells, IFN-α is predominantly produced in hematopoietic cells such as monocyte/macrophages and dendritic cells, especially the plasmacytoid dendritic cells. IFN-γ is induced in response to different extracellular signals, including interleukins IL2, IL12, and IL18, preferentially, in NK and T cells (Green et al., 2017). These cytokines activate JAK/STAT, NF-κB, JNK, ERK and p38 MAPK pathways, depending on the cell line, and induce transcription of IFN-γ in response to NFAT, NF-κB, STAT and AP1 transcription factors.

IFN-α and -β bind to the IFN-α/β receptor (IFNAR) in an autocrine and paracrine manner, triggering the induction of the Janus kinase/signal transducers and activators of transcription (JAK/STAT) signaling cascade (**Figure 2**) (Hertzog and Williams, 2013; Ivashkiv and Donlin, 2014; Schneider et al., 2014). The outcome is recruitment, followed by phosphorylation, dimerization and finally nuclear translocation of STAT1 and STAT2. Once in the nucleus, the complex of STAT1 and STAT2 is bound to IRF9/p48, forming the IFN-stimulated gene factor 3 (ISGF3) complex which induces the transcription of hundreds of IFN-stimulated genes (ISGs) (**Figure 2**) (Hertzog and Williams, 2013; Ivashkiv and Donlin, 2014; Schneider et al., 2014). In a highly analogous manner, IFN-γ, the sole member of the type II IFN family, binds the IFN-γ receptor and activates signaling through the JAK/STAT pathway resulting in STAT1 phosphorylation followed by nuclear translocation (Hu and Ivashkiv, 2009). Once activated, STAT1 binds the gamma-activated sequence (GAS) close to its target genes as a homodimer, triggering the transcriptional induction of STAT1-regulated genes. As mentioned above, the majority of IFN-γ-regulated genes are also induced by type I IFNs and thus, type I and type II IFN response show significant overlap in terms of the downstream effects (Hu and Ivashkiv, 2009; Pollard et al., 2013).

The final outcome of the IFN signaling is the induction of ISGs, many of which exert antimicrobial activity via regulation of different steps of cellular gene expression in addition to direct antimicrobial effects. For example, several ISGs including IFIT-1, IFIT-2, IFITM3, ISG15, ISG20, RNase L, PKR, viperin and BST2/Tetherin are known to have antiviral activity (Randall and Goodbourn, 2008). Thus, activation of the IFN response results in the induction of a cell-intrinsic antimicrobial state in both the infected and the neighboring cells which limits the spread of infectious agents. Further, these potent cytokines also affect both the innate and adaptive immune response through promoting antigen presentation, natural killer cell function, production of antibody in B cells and T cell effector function (Ivashkiv and Donlin, 2014). As mentioned above, most cells are able to launch the type I IFN response, however, cell type and context have a strong effect on the magnitude of the induced response and the specific subsets of effector genes activated (Hertzog and Williams, 2013; Ivashkiv and Donlin, 2014; Schneider et al., 2014). On the other hand, the IFN response itself is subject to negative feedback regulation by a number of ISGs and other cellular signaling pathways (Yoshimura et al., 2007; Hertzog and Williams, 2013; Ivashkiv and Donlin, 2014; Schneider et al., 2014; Porritt and Hertzog, 2015).

While the IFN cascade is mainly activated in response to the presence of exogenous viral RNAs, a group of long non-coding cellular RNAs, namely the retrotransposon-derived transcripts, also activate the IFN response (Yu Q. et al., 2015). It has been shown that activation of the Long Interspersed Element-1 (LINE-1) retroelements results in induction of the expression of IFN-β and ISGs (Yu Q. et al., 2015). Prior treatment of cells with IFN leads to suppression of LINE-1 replication, and mutations that inactivate different steps of the IFN signaling pathway cause an increase in LINE-1 replication (Yu Q. et al., 2015). Although the mechanism behind the induction of the IFN response by LINE-1 replication is not known, it is likely that the replication state of LINE elements and other retrotransposons are detected by either the same or similar sensors to those that detect the presence of exogenous viral RNA in the cell. Existing data suggest that the replication of another class of retrotransposons, the Small Interspersed Elements (SINEs) similarly results in activation of the IFN response (Leonova et al., 2013). Interestingly, it has been suggested that LINE-1 elements may contribute to the pathogenesis of autoimmune disorders such as Systemic Lupus Erythematosus (SLE) (Crow, 2010; Nakkuntod et al., 2011), which is known to involve activation of the IFN response (Bronson et al., 2012; Elkon and Wiedeman, 2012). Additional studies of the connection between autoimmunity and the replication state of cellular repeat elements will likely provide novel insight into the mechanistic basis of development of autoimmune diseases.

## lncRNAs as Crucial Regulatory Factors in the IFN Response

A number of high throughput transcriptomic analyses in both human and mouse have revealed the presence of strong changes in the expression of lncRNAs during the IFN response (Peng et al., 2010; Carnero et al., 2014; Josset et al., 2014; Kambara et al., 2014, 2015; Barriocanal et al., 2015). In the mouse studies, which were performed in both mouse lung tissue and cultured mouse embryonic fibroblasts, a significant fraction of the differentially expressed genes belonged to lncRNAs, which were either up or down-regulated after interferon stimulation and infection with respiratory viruses (Peng et al., 2010; Josset et al., 2014). Interestingly, promoter analysis and expression correlation studies raised the possibility that a significant fraction of the induced lncRNAs may be novel ISGs that were directly induced through the IFN signaling cascade (Peng et al., 2010; Josset et al., 2014).

The results obtained in the murine models were closely analogous to those obtain using human cells. In one such study, primary human hepatocytes from five donors of different ages and genders were subjected to high throughput transcriptome analysis before and at three time points of 3, 9, and 24 h after treatment with IFN-α (Kambara et al., 2014). Similar to what had been observed in the mouse, IFN stimulation resulted in both induction and repression of expression of a large number of lncRNAs. Many of the induced lncRNAs were likely direct targets of the JAK-STAT signaling pathway and thus, novel ISGs. While the majority of the induced lncRNAs remained upregulated for the three time points analyzed in this study, a fraction of them showed a shorter duration of induction and were limited to the earlier time points of the study (Kambara et al., 2014).

A third set of high throughput studies in HuH7 human hepatocytes focused on changes in gene expression observed at later time points (after 72 h) of treatment with a high dose of IFN-α2 using both microarray analysis (Carnero et al., 2014) and RNA-seq (Barriocanal et al., 2015). Interestingly, similar to the results of the study by Kambara et al. (2014), the lncRNAs that showed differential expression were almost equally divided between upregulated and downregulated ones in both microarray and RNA-seq-based datasets, while almost all differentially expressed protein coding genes were upregulated. In addition, both sets of studies revealed a significant number of differentially expressed lncRNAs which were either vicinal to or overlapped protein-coding genes that function in the immune response (Carnero et al., 2014; Kambara et al., 2014, 2015; Barriocanal et al., 2015). As mentioned above, in some cases the function of a lncRNA depends on its genomic locus and thus, these studies potentially point to the presence of an IFN-activated regulatory network of lncRNAs which may function in fine-tuning the immune response.

### Regulation of the Expression of IFNs by lncRNAs

Induction of transcription of IFN genes by external or internal stimuli constitutes a key step in the IFN response and is known to be regulated by several protein-mediated mechanisms (Hertzog and Williams, 2013; Ivashkiv and Donlin, 2014; Schneider et al., 2014). Interestingly, recent evidence point to the presence of a number of lncRNA-mediated regulatory mechanisms acting on this step. At the IFNG/IFN-γ locus, lncRNA IFNG-AS1 (IFNG-antisense-1), also known as Tmevpg1 (Vigneau et al., 2003; Collier et al., 2012; Collier, 2014) and NeST (Gomez et al., 2013), is located downstream of the IFNG genic region and in human, overlaps this locus. The expression of IFNG-AS1, which is present in CD4+ and CD8+ T cells in addition to natural killer (NK) cells, shows a strong positive correlation with that of IFNG (Vigneau et al., 2003; Collier et al., 2012; Collier, 2014). The expression of IFNG-AS1 contributes to the induction of IFNG expression in CD4+ cells during their differentiation into Th1 polarization (Collier et al., 2014) and in CD8+ T cells after stimulation with PMA (phorbol 12-myristate 13-acetate) and ionomycin (Gomez et al., 2013). Mechanistic studies have shown that IFNG-AS1 acts in *trans* via interacting with WDR5, a subunit of the MLL/SET1 histone H3 lysine 4 methyltransferase complex, potentially recruiting the complex to the IFNG locus to change its methylation state (Gomez et al., 2013).

A more recent study in human trophectoderm progenitors suggested that knock down of a novel lncRNA, lncRHOXF1, which is expressed in trophectoderm and primitive endoderm cells in human blastocyst-stage embryos, may lead to upregulation of MDA5, RIG-I and IFN-β (Penkala et al., 2016). While the mechanism of the observed gene expression changes is not yet determined, the above data suggest a repressive role for lncRHOXF1 in the IFN response. NEAT1, a well-studied lncRNA with a structural role in paraspeckles, also regulates IFN-β production and RIG-I function, albeit in the opposite direction (Ma et al., 2017). Studies in the human umbilical vein endothelial cells indicated that upon infection with Hantaan virus, transcription of NEAT1 was induced through activation of RIG-I/IRF7 pathway. NEAT1, in turn, mediated the relocation of the splicing factor proline- and glutamine-rich protein (SFPQ) to paraspeckles, thus removing its transcriptional inhibitory effect on the expression of RIG-I and DDX60 leading to IFN-β production (Ma et al., 2017). In this manner, NEAT1 acts as a positive feedback regulator of RIG-I activation during viral infections. It is likely that future studies will reveal additional lncRNAs which regulate the signaling steps upstream of the expression of IFN genes, thus acting as global regulators of the IFN response.

### lncRNAs Governing the Expression of IFN-Stimulated Genes

As mentioned above, high throughput transcriptomic studies have revealed the presence of a large number of lncRNAs that show differential expression in response to IFN stimulation (see **Table 1** for some functionally studied examples). One such RNA, which was identified in primary human hepatocytes by Kambara et al. (2014), originated from a locus downstream of the protein-coding ISG CMPK2 and showed a strong upregulation after IFN stimulation in additional cell types from both human and mouse. The induction of this RNA, similar to protein-coding ISGs, was dependent on the JAK-STAT signaling pathway. Knock down studies on this lncRNA, which was named lncRNA-CMPK2/NRIR (Negative Regulator of the IFN Response), resulted in a strong reduction in HCV replication in IFN-stimulated hepatocytes. Additional analyses indicated that knockdown of NRIR led to upregulation of both basal and IFN-stimulated transcription of a number of protein-coding antiviral ISGs, which could be best explained by loss of transcriptional inhibition in knockdown cells. While the mechanism of function of NRIR has not been determined, its nuclear localization together with its ability to affect the transcription of its target genes were consistent with either an epigenetic or transcriptional regulatory mechanism. Together, these data provided evidence for the presence of a lncRNA-mediated negative regulatory mechanism during the IFN response (Kambara et al., 2014). As NRIR and likely many other unstudied lncRNAs that regulate the IFN response are themselves bona-fide ISGs, it is plausible that at least some such lncRNAs may affect their own expression in addition to that of other target genes, thus creating an additional layer of self-regulatory loops. However, to our knowledge this possibility has remained unstudied.

**Table 1:** Summary of the mode of induction and action of the lncRNAs involved in regulation of the IFN response.

lncRNA	Known mode of induction	Known mechanism of induction	Locus	Known mode of action	Known mechanism of action
NRIR (lncRNA-CMPK2)	Induced by IFN-α, β and γ	JAK-STAT pathway (ISG)	hg38 chr2:6,828,880–6,833,531	Acts in trans	Transcriptional downregulation of several target genes including other ISGs
NRAV	Downregulated after infection with influenza and other viruses	Not known	hg38 chr12:120,490,328–120,495,940	Acts in trans	Downregulation of several target genes including other ISGs through binding ZONAB
BISPR	Induced by IFN-α, β and γ	JAK-STAT pathway (ISG)	hg38 chr19:17,405,743–17,415,738	Acts in cis/trans	Transcriptional regulation of expression of its promoter-sharing ISG neighbor BST2
EGOT	Induced by IFN-α, poly(I:C) or infection with HCV, SFV or inflyuenza virus	NF-κB pathway	hg38 chr3:4,749,192–4,751,590	Acts in cis	Transcriptional downregulation of several target genes including other ISGs
LUARIS	Downregulated after IFN-β or poly(I:C) treatment	IRF3-mediated downregulation	hg38 chr7:43,508,728–43,522,542	Acts intrans	Through binding to ATF2 positively regulates the expression of several target genes including other ISGs
IFNG-AS1 (Tmevpg1, NeST)	Th1 CD4+ T cell development	Induced by TBX21 (TBET), NF-kB and ETS1	hg38 chr12:67,989,529–68,234,686	Acts in trans	Changes the methylation status of IFNG locus through interacting with WDR5, leading to induction of IFNG gene
lncRHOXF1 (RHOXF1P1)	Not known	Not known	hg38 chrX:120,010,717–120,015,551	Not known	Negative regulation of expression of MDA5, RIG-I and IFN-β
NEAT1	Induced upon Hantaan virus infection	RIG-I/IRF7 pathway	hg38 chr11:65,422,798–65,445,540	Acts in trans	Sequesters SFPQ, leading to induction of RIG-I and DDX60 and thus, IFN-β production.

Shortly after the discovery of NRIR, another lncRNA named NRAV (Negative Regulator of Antiviral Response) was described in a study focusing on genes differentially expressed in response to influenza virus H1N1 infection in A549 human alveolar epithelium cell line (Ouyang et al., 2014). NRAV originated from what is likely a bidirectional promoter that also gave rise to the main isoform of the dynein light chain gene DYNLL1 and was both spliced and polyadenylated. The level of NRAV was markedly reduced following infection with influenza virus and a number of other viruses in several cell lines. Importantly, forced overexpression and knock down experiments revealed that the expression level of NRAV directly correlated with the extent of viral reproduction. A microarray study of NRAV-overexpressing cell lines showed the reduction of the level of a significant number of ISGs, and additional experiments indicated that NRAV, similar to NRIR (Kambara et al., 2014), has the ability to partially block induction of the expression of its target ISGs in response to IFN stimulation (Ouyang et al., 2014).

Another lncRNA with a negative regulatory function in the IFN response is EGOT (Eosinophil Granule Ontogeny Transcript). EGOT is a structured polyadenylated nuclear lncRNA conserved, at least, in all placental mammals (Rose and Stadler, 2011). Interestingly, EGOT genomic locus shows enhancer marks with high histone 3 lysine 4 monomethylation and low trimethylation, indicating that EGOT could be an enhancer RNA (Heintzman et al., 2007). EGOT overlaps an intron of the inositol 1,4,5-trisphosphate receptor 1 (ITRP1) gene in antisense orientation but EGOT depletion does not affect ITRP1 cellular levels (Prior et al., unpublished observation). EGOT was first described as a lncRNA expressed in eosinophils during development and maturation and is thought to function in mature eosinophils in regulating the levels of toxic molecules, such as the major basic protein and the eosinophil derived neurotoxin (Wagner et al., 2007). However, GTEx studies show that the highest levels of EGOT are found in non-hematopoietic tissues such as breast, vagina, pancreas, pituitary and kidney cortex (GTEx Consortium, 2013). A recent study in HuH7 cells indicated that the level of EGOT shows a dramatic increase after infection with HCV and other RNA viruses (see below) (Carnero et al., 2016). EGOT was also induced in response to very high doses of IFN-α, but at much lower levels compared to what is observed after infection with RNA viruses. Knock down of EGOT in HCV infected cells led to an increase in the expression of a subset of ISGs including GBP1, ISG15, Mx1, BST2, ISG56, IFI6, and IFITM1, resulting in reduced viral replication (Carnero et al., 2016). Taken together, these results indicate that although EGOT is not a bona fide ISG itself, it is yet another lncRNA with a negative regulatory impact on ISG induction and thus, the IFN response.

Considering the rather small number of lncRNAs that have been studied in the context of the IFN response, the discovery of the negative regulatory roles of NRIR, NRAV and EGOT on the transcriptional induction of ISGs suggests that lncRNAs may play a prominent role in negative feedback loops controlling the IFN response and possibly other signaling cascades in the immune response. Interestingly, several protein factors involved in negative regulation of IFN response have been identified (Yoshimura et al., 2007; Hertzog and Williams, 2013; Ivashkiv and Donlin, 2014; Schneider et al., 2014; Porritt and Hertzog, 2015). Defining the interaction of the negative regulatory lncRNAs with the positive and negative regulatory proteins in the context of IFN response will yield a unified picture of the feedback mechanisms which mediate the termination of IFN signaling cascade.

While the above described lncRNAs had a negative regulatory impact on the IFN response, a recent study has provided evidence for lncRNA-mediated positive regulation of the IFN cascade. A screen for IRF3-dependent genes in HuS immortalized human hepatocytes led to the identification of an annotated but unstudied lncRNA (LOC100506895/AC011738.4/ENST00000436105.1) that was down-regulated in an IRF3-dependent manner after poly(I:C) treatment (Nishitsuji et al., 2016). This transcript, which was dubbed lncRNA#32 and was later renamed LUARIS (lncRNA upregulator of antiviral response interferon signaling), overlaps introns 21 and 22 and exon 22 of the protein-coding gene HECW1 in antisense orientation (hg38 chr7:43,508,728-43,522,542). Unlike NRIR, the level of LUARIS was reduced after stimulation with IFN-β. Interestingly, both in the presence and absence of IFN stimulation, knock down and overexpression of LUARIS led to dramatic reduction and upregulation of expression of several ISGs, respectively. Indeed, forced overexpression of LUARIS led to suppression of replication of a number of viral pathogens including HCV, further proving that it acts as a positive regulator of the IFN response (Nishitsuji et al., 2016). Mechanistic studies indicated that the transcriptional stimulatory action of this lncRNA was likely mediated through its binding to the activating transcription factor 2 (ATF2) (Nishitsuji et al., 2016). Why should a positive regulator of the IFN response be downregulated by IFNs? It is likely that the expression level of LUARIS is used to adjust the magnitude of the IFN response through the action of multiple regulatory pathways that control its transcription or stability. Defining the identity of additional signaling pathways that control the expression of this and other similarly acting lncRNAs will provide crucial insights into the complex network of interactions that regulate the antiviral response.

While the lncRNAs described above regulated a number of ISG targets, another IFN-induced lncRNA named BISPR (BST2 IFN-Stimulated Positive Regulator) seems to affect the expression of a single target gene. Independent studies from two groups (Carnero et al., 2014; Kambara et al., 2014, 2015; Barriocanal et al., 2015) identified BISPR as a lncRNA that was induced in response to IFN-α stimulation through the JAK/STAT pathway in multiple cell lines including the THP1 monocytes (Kambara et al., 2015) and HuH7 human hepatocytes (Carnero et al., 2014; Barriocanal et al., 2015). BISPR originated from a bidirectional promoter that also gave rise to BST2/Tetherin, a well-studied protein-coding ISG. RNAi-mediated knockdown of BISPR reduced the IFN-mediated induction of BST2 expression (Barriocanal et al., 2015; Kambara et al., 2015) pointing to the potential presence of a regulatory mechanism for coordinating the expression of BISPR with that of its promoter sharing gene BST2. Interestingly, after stimulation by IFN-α, the increase in cellular level of BISPR preceded the rise in the level of BST2, suggesting that expression of BISPR either induced or facilitated the initiation of transcription of BST2 (Kambara et al., 2015). Confirming these findings, forced overexpression of the spliced BISPR RNA from a transgene led to up-regulation of BST2, indicating that BISPR controls BST2 expression via interactions mediated through the BISPR RNA itself, rather than by impacting the local chromatin environment through its transcription (Kambara et al., 2015). As a number of protein-coding ISGs and immunity-related protein-coding genes originate from bidirectional promoters which also give rise to lncRNAs, it is plausible that a regulatory mechanism similar to the one described above may be present in at least a subset of them. Taken together, despite the small number of studied lncRNAs, existing data points to a critical role for this class of transcripts in regulation of the IFN response and the antiviral activity against human pathogens such as the hepatitis C virus, which is a well-studied disease model for the impact of IFNs on human pathogens. Future studies are likely to identify many additional lncRNAs that regulate different steps of this key aspect of the innate immune response.

## Interaction of the Non-Coding Transcriptome and RNA Viruses: the Example of Hepatitis C Virus

Hepatitis C (HCV) is a deadly virus that affects ∼2% of the world’s population (60–170 million people) (Manns et al., 2017). About 500,000 people die every year from HCV-related diseases (Webster et al., 2015; Bukh, 2016). After the initial infection, most patients develop an asymptomatic chronic infection that causes liver damage and therefore, may progress to liver steatosis, fibrosis, cirrhosis and hepatocellular carcinoma (HCC). In the course of 30 years of HCV infection, ∼20% of patients will develop cirrhosis. In fact, HCV infection is nowadays one of the major inducers of liver cirrhosis. As HCV infection induces the expression of several oncogenic factors and liver cirrhosis is a niche for HCC, ∼3–7% of cirrhotic patients will develop liver tumors every year (Llovet and Villanueva, 2016).

In spite of the efforts of many groups, an effective vaccine to prevent HCV infection has not yet been developed. Recently, several inhibitors that target different viral proteins have been approved for the treatment of HCV infection (D’Ambrosio et al., 2017). The efficacy of these treatments grazes a complete sustained viral response, allowing the claim that HCV infection can be cured (Bourlière et al., 2017). A fraction of the small number of patients that resist the treatment and do not show viral clearance have high levels of liver cirrhosis. Some of the patients with HCV infection and HCC show a transient response with a later relapse of the virus, probably because HCC architecture and/or composition impairs drug penetration and serves as a reservoir for infected cells (Prenner et al., 2017). Some authors have observed an increased risk of HCC recurrence in patients that have cleared HCV infection (Llovet and Villanueva, 2016; Reig et al., 2016). In spite of this, there are good reasons for optimism. After sustained viral responses, liver fibrosis can regress, the risk of cirrhosis-related complications, including HCC, is reduced and the overall survival of the patients increases (van der Meer and Berenguer, 2016; Nahon et al., 2017; van der Meer et al., 2017). However, there is still a long way to achieve the goal of HCV cure. Patients cured of chronic HCV infection are at risk for reinfection if exposed. Work toward the development of an effective vaccine to prevent HCV infection must be intensified to cure the disease (Bukh, 2016). Research about HCV infection should not stop and a rigorous epidemiological follow up should be carried out given the high prevalence of HCV infection and the ability of HCV to generate escape mutants resistant to the treatments (Ramirez et al., 2016).

### HCV Infection

The seven genotypes of HCV share similar characteristics. HCV viral particle is enveloped, small (40–80 nm) and encloses the genome coated by the core protein (Gastaminza et al., 2010; Catanese et al., 2013). The genome is an RNA molecule of 9.6 Kb of length and positive polarity that may function as messenger RNA (mRNA). Multiple coding regions of the viral genome and the 5′ and 3′ untranslated regions (UTRs) are highly structured, well-conserved and required for replication and encapsidation (Piñeiro and Martinez-Salas, 2012; Pirakitikulr et al., 2016; Shi et al., 2016). The structure located at the 5′ end contains an internal ribosome entry site (IRES) that allows viral RNA cap-independent translation. Translation synthesizes a polyprotein that can be cleaved by viral and cellular proteases co- or post-transcriptionally. Cleavage releases three major structural proteins (core, and the two envelope glycoproteins, E1 and E2) and seven non-structural proteins (p7, NS2, NS3, NS4A, NS4B, NS5A, and NS5B). These proteins are required for polyprotein cleavage, cellular antiviral response blockade, viral replication, assembly and release (Carnero and Fortes, 2016).

The majority of HCV virions are transported in the blood embedded into very low or low-density lipoproteins (VLDLs and LDLs) formed by triglycerides, apolipoproteins and cholesterol or phospholipids (Bassendine et al., 2013). This coat may help virions to escape from neutralizing antibodies and aids hepatocyte infection. LDL Receptor (LDLR), claudin-1 (CLDN1) and occludin (OCLN) are some of the several receptors identified to play a role in viral entry into hepatocytes (Ploss et al., 2009; Zeisel et al., 2011). After endocytosis, the virion uncoats in response to the acidic pH of the endosome by fusing the viral envelope with the endosome and releasing the viral genome into the cytoplasm. Viral RNA translation occurs in the rough endoplasmic reticulum (ER). There, a membranous web (MW) is formed by double-membrane vesicles, the replication complex is assembled and viral replication takes place (Romero-Brey et al., 2012; Meyers et al., 2016). Replication is carried out by NS5B, the viral RNA-dependent RNA polymerase. The positive-stranded RNAs are used as templates to produce negative-strand RNAs which are then used as guides to synthesize large quantities of new viral genomes. The low fidelity of HCV RNA-dependent RNA polymerase produces a highly variable progeny (Geller et al., 2016). Thus, in every infected patient, the incoming viruses generate a collection of descendants with related but non-identical HCV genomes, known as quasi-species (Martell et al., 1992).

The new viral genomes can be used for translation of new viral proteins, replication to produce new viral genomes or packaging into new viral particles. Assembly takes place close to lipid droplets (LDs) bound to ER membranes. NS5A molecules from the replication complex bind simultaneously to the newly synthesized RNA and core protein, helping the interaction between core protein and viral RNA which will lead to formation of a nucleocapsid bound to a LD (Masaki et al., 2008). The envelope is acquired after budding through the ER and the particle is released bound to LDL or VLDLs through the secretory pathway (Lindenbach and Rice, 2003; Bayer et al., 2016; Syed et al., 2017). Thus, inhibition of the synthesis of lipid components blocks viral assembly (Bassendine et al., 2013).

### HCV and the Antiviral IFN Response

#### HCV-Infection and Protein Coding Genes That Regulate the IFN Response

Successful replication requires inactivation of the antiviral response, which is initiated soon after infection (Loo et al., 2006; Saito et al., 2008). RIG-I canonical sensor may recognize the incoming viral genome. Non-canonical sensors such as protein kinase R (PKR) and the DEAD box helicase DDX3X also recognize the 5′UTR and the 3′UTR of HCV genome, respectively (Arnaud et al., 2010, 2011; Li et al., 2013). Later, RIG-I and TLR3 can sense viral dsRNAs produced during replication (Binder et al., 2011; Li et al., 2012). Sensor activation induces NF-κB and IRFs via MAVS and TRIF and the synthesis of several subtypes of IFN (**Figure 2**). While type III IFNλ signaling has a special impact on HCV infection (Ge et al., 2009; Thomas et al., 2009; Boisvert and Shoukry, 2016), type I IFNα has been traditionally used to cure the disease (McHutchison et al., 1998). IFN signaling is a potent activator of the expression of several ISGs that limit HCV replication. They function by reinforcing IFN signaling (STAT1, STAT2, IRF1, 3, 7 and 9, PKR, OAS or RNase L) and blocking every step of the viral cycle: viral entry (IFITM, TRIM, Mx, CH25H), RNA replication, translation and stability (OAS, IFIT, GBP1), assembly and release (tetherin/BST2, viperin) (Carnero and Fortes, 2016). Viperin binds to NS5A in the replication complex and the LDs, blocking NS5A function in viral replication and assembly (Helbig et al., 2005, 2011). BST2 or tetherin impedes the budding of viral particles by attaching virions to the cell surface and allowing their degradation by the lysosomes (Neil et al., 2008; Dafa-Berger et al., 2012). Interestingly, a strong antiviral response is only achieved by the cooperative function of several ISGs and not by their individual actions (Metz et al., 2012).

To combat the antiviral response, HCV has evolved to express viral proteins that block IFN synthesis and signaling and interfere with the functionality of antiviral molecules. NS3-NS4A protease cleaves MAVS and TRIF impeding sensor signaling (Foy et al., 2005; Li et al., 2005; Meylan et al., 2005). In addition, RIG-I signaling may be affected by HCV-mediated induction of autophagy (Ke and Chen, 2011). NS4A/B precursor blocks MHC Class I transport to the cell surface. Finally, core protein blocks IFN signaling by upregulating the expression of SOCS3 or the protein phosphatase PP2Ac, which are negative regulators of STAT1 transcription factor (Bode et al., 2003; Duong et al., 2004; Kawaguchi et al., 2004; Walsh et al., 2006).

The blockade of IFN signaling induced by the virus explains why many patients fail to respond to IFN treatment (Chen et al., 2005; Sarasin-Filipowicz et al., 2008). Surprisingly, many non-responder patients have increased ISG mRNA levels both in infected and uninfected hepatocytes (Hedegaard et al., 2017). One way to explain why these patients fail to respond to IFN in spite of expressing high levels of ISG mRNAs is that these mRNAs are not efficiently translated into antiviral proteins. Interestingly, it is also possible that some ISG mRNAs are translated into proteins that exert proviral functions in HCV infected cells. Both possibilities may turn out to be correct. DsRNA-activated PKR phosphorylates eukaryotic translation initiation factor eIF2α, leading to inhibition of cap-dependent translation. This does not affect HCV protein translation, which is IRES-mediated and eIF2α-independent (Garaigorta and Chisari, 2009). However, translation of ISG mRNAs is likely to be drastically affected, leading to HCV-infected cells with high levels of ISG mRNAs that are not translated into antiviral proteins. Therefore, PKR is an ISG with a proviral function in HCV infection. Other proviral ISGs include those that function as negative regulators of the antiviral response allowing cells to return to homeostasis after IFN induction and non-canonical sensors such as DDX3X. After viral genome sensing, DDX3X activates IKKα, which induces the expression of lipogenic genes essential for viral assembly (Li et al., 2013; Pène et al., 2015).

A paradigm for an ISG that promotes HCV replication is ISG15 (Broering et al., 2010). ISG15 is an ubiquitin-like moiety cotranslationally attached to proteins by the IFN-induced ISGylation machinery. Therefore, after HCV infection, the IFN response induces ISGylation of new proteins, which are viral proteins and cellular antiviral factors (Durfee et al., 2010). ISGylation may affect the functionality of targeted proteins by modifying their structure and/or stability. IRF3 is stabilized by ISGylation-mediated inhibition of polyubiquitination (Shi et al., 2010). However, RIG-I ISGylation blocks ubiquitination and functionality, leading to decreased levels of IFN and increased replication of HCV (**Figure 2**) (Kim et al., 2008; Broering et al., 2010). Therefore, in HCV infection, ISG15 functions as a proviral ISG. In fact, upregulation of ISG15 is associated with poor response to IFN treatment and poor prognosis in HCV-infected patients (Chen et al., 2011).

The combined action of PKR and the ISGylation and ubiquitination pathways in HCV-infected cells results in decreased cap-dependent protein translation and increased protein modification that leads to protein malfunction and destabilization. Under such a protein-hostile environment it must be challenging to maintain proper cell functionality and virus replication. Therefore, it is conceivable that both cells and viruses have evolved to achieve functionality under these conditions through the expression of functional non-coding RNAs, which are at least partially immune to protein-hostile conditions (Fortes and Morris, 2016).

#### Effects of HCV Infection on the Non-coding Genome

Hepatitis C virus infection causes a deregulation in the expression of the non-coding genome, which may function either to facilitate or to block viral viability. This has been best studied for miRNAs. Several cellular miRNAs have been described to target the HCV genome to help or prevent viral replication or to regulate the expression of cellular factors required for the virus cell cycle or the antiviral response. Generally, virus replication-related factors induce proviral and reduce antiviral miRNAs while the innate immune response plays the opposite effect. The interplay of HCV infection and miRNAs has been reviewed elsewhere (Singaravelu et al., 2014, 2015).

In contrast, the world of infection-induced lncRNAs is widely unexplored and HCV-related lncRNAs are not an exception. The few studies performed clearly indicate that, similar to what has been described for miRNAs, several cellular lncRNAs are deregulated in response to HCV replication or to the antiviral response induced by viral infection (Barriocanal and Fortes, 2017). The function of some of them has been studied. These lncRNAs affect HCV replication by regulating cell metabolism, proliferation and the antiviral response. HCV could also express viral lncRNAs generated by XRN1 exonuclease-mediated 5′ to 3′ degradation of the viral genome. These subgenomic RNAs have lost the initial sequences of the IRES and therefore they most likely fall into the category of lncRNAs (Moon et al., 2015).

Studies performed so far to identify HCV-deregulated cellular lncRNAs have employed infected cultured cells or liver tissue from infected patients. In the latter case, it is difficult to establish whether the deregulation of the lncRNAs indeed results purely from HCV infection, as the evaluated tissue has also developed liver cirrhosis and/or HCC. Comparison of the level of lncRNAs in these tissues versus healthy liver identifies the lncRNAs that are deregulated by HCV infection, liver cirrhosis or HCC alone or in all possible combinations. In line with this, some of the lncRNAs deregulated in HCV-infected livers have been shown to play a role in the development of liver cirrhosis and/or HCC (Yuan et al., 2014; Fu et al., 2017). This has been recently reviewed (Barriocanal and Fortes, 2017). Other lncRNAs studied in patients with HCV-derived HCC could be bona-fide HCV-induced lncRNAs. This is the case for UCA1, which is also upregulated in liver and serum of patients with HCC. UCA1 serum levels correlate significantly with HCV antibodies and increase in tissue culture cells infected with HCV (Carnero et al., 2016; Kamel et al., 2016; Barriocanal and Fortes, 2017).

Infection with HCV or expression of the core protein in cultured cells has led to the identification of additional HCV-induced oncogenic lncRNAs such as PVT1, CASC15 and HOTAIR (Carnero et al., 2016; Li et al., 2016). The induction of lncRNA HOTAIR by the core protein may in turn lead to increased viral replication by silencing SIRT1 promoter and affecting glucose and lipid metabolism (Li et al., 2016). HCV-induced lncRNAs such as UCA1, PVT1 or CASC15 are upregulated in response to viral replication and not after activation of the antiviral response. Thus, these lncRNAs do not increase when cells are treated with IFN or pathogen associated molecular patterns (PAMPs) such as poly(I:C) or LPS, or in cells infected with other viruses such as HBV, influenza, adenovirus or Semliki Forest Virus (SFV), which replicate in the nucleus or in the cytoplasm, have DNA or RNA viral genomes and lead to acute or chronic infections (Carnero et al., 2016). Instead, it has been proposed that HCV replication may induce specific pathways for the activation of these lncRNAs. For example, viral protein NS5A induces MYC, which in turn activates PVT-1 transcription (Carramusa et al., 2007). Similarly, HCV-induced reactive oxygen species (ROS) is likely to induce the activation of the expression of UCA1 in HCV infected cells. Indeed, ROS inhibits C/EBPα, a negative regulator of UCA1, and stabilizes the UCA1 inducer HIF-1α, leading to UCA1 induction (Miura et al., 2008; Nishina et al., 2008).

On the other hand, several lncRNAs have been described that are upregulated both in HCV-infected cells and in cells treated with IFN or PAMPs such as poly(I:C), or when cells are infected with viruses different than HCV. Therefore, they can be considered to be lncRNAs upregulated by the antiviral response.

As described above, several IFN-induced lncRNAs have been identified after comparing the transcriptome of cells treated with IFN and controls (Carnero et al., 2014; Kambara et al., 2014, 2015; Barriocanal et al., 2015). This is the case for NRIR and BISPR (see above), ISR2 (IFN-stimulated lncRNA2), ISR8 and lncISG15 (Carnero et al., 2014; Barriocanal et al., 2015). Interestingly, the loci of the above lncRNAs are neighboring those of ISGs that play a key role in HCV infection (CMPK2 and Viperin, BST2, GBP1, IRF1 and ISG15, respectively). As detailed above, NRIR, which is a negative regulator of the IFN response, is significantly upregulated in the liver of HCV-infected patients compared to healthy controls (Kambara et al., 2014). Therefore, HCV may use NRIR to increase replication. Similarly, ISR2, ISR8, lncISG15 and BISPR are upregulated in liver and cultured cells infected with HCV compared to controls (Carnero et al., 2014; Barriocanal et al., 2015). Unexpectedly, HCV-induction of ISR2 and ISR8 is higher than induction with other viruses such as influenza, adenovirus or SFV, or mutant versions that allow induction of a strong antiviral response (Carnero et al., 2014). This suggests that HCV may have an additional mechanism distinct from the IFN response for inducing the expression of these lncRNAs. Although their cellular function has not yet been described, guilt-by-association studies predict that ISR8 is an antiviral factor that induces the immune system and the antiviral response and ISR2 regulates the action of antiviral sensors and IFN activation (Carnero et al., 2014). In the case of BISPR, increased expression by IFN leads to higher levels of BST2 and decreased virion release (Neil et al., 2008; Dafa-Berger et al., 2012; Barriocanal et al., 2015; Kambara et al., 2015). Thus, induction of IFN-induced lncRNAs may have positive effects (in the case of NRIR) or negative effects (in the case of BISPR) on HCV replication.

STAT3 transcription factor, a negative regulator of the IFN pathway, can be induced by IFN, growth factors, stress, several cytokines such as IL6 and by HCV infection (Wang et al., 2011). HCV core and other viral proteins induce ROS and activate STAT3 by several mechanisms (Yoshida et al., 2002; Waris et al., 2005; Machida et al., 2006). STAT3 activation, in turn, favors viral replication by blocking type I IFN pathway and through positive regulation of microtubule dynamics (McCartney et al., 2013). More recently, it has been shown that several lncRNAs are induced by STAT3 activation, including lncIGF2-AS and lnc7SK, which help MW formation by increasing the level of phosphatidylinositol 4-phosphate kinase (Xiong et al., 2015). Therefore, in this manner, both the IFN response and HCV infection benefit viral replication via STAT3-mediated induction of expression of lncRNAs.

Finally, a group of lncRNAs are induced 3–30 fold by IFN or PAMPs such as poly(I:C) and up to 100 times more by HCV infection (Carnero et al., 2016) (**Table 2**). These lncRNAs were identified by transcriptome analysis of cultured liver cells with and without HCV infection and were named CSRs, after HCV Stimulated lncRNAs.

**Table 2:** List of lncRNAs induced by HCV and poly(I:C).

Name	Position	Name	Alternative name	Fold induction by IFN	Fold induction by pl:C	Fold induction by HCV
CSR3	chrl2:93936239-93965544R	S0CS2-AS1	lnc-UBE2N-2		2.6	64
CSR6	chrl5:95819690-95832714R	CTD-253611.1	lnc-AC016251.1-8		6.7	24
CSR7	chrl7:70399463-70588479R	LINC00673	Inc-SLC39A11-1		3.6	34
CSR20	chr6:53493178-53496192F	RP11-345L23.1	LINC01564 lnc-LRRCl-3		16.2	111
CSR31	chr2:97163383-97173846R	NEURL3		12.0	30.8	1941
CSR32	chr3:4790876-4793274R	EGOT	lnc-AC018816.3.1	3.5	6.2	797

*The genomic position, name and alternative names are indicated. The fold induction has been quantified in cells treated with IFN, poly(I:C) or in cells infected with HCV versus untreated cells*.

As mentioned above, the highest upregulation of these CSRs has been observed when cells are infected with HCV. However, it has been shown that infection with other viruses also leads to the induction of their expression. DNA viruses such as adenovirus and RNA viruses such as influenza, SFV, HCV or mutant versions of influenza that allow IFN induction, upregulate CSR3, 7 and 31. On the other hand, CSR6 is only induced by adenovirus, CSR20 by influenza virus infections. Similarly, CSR32/EGOT is upregulated in response to RNA viruses (HCV, influenza, SFV) but not DNA viruses (adenovirus or HBV). Existing data suggest that the expression of EGOT (see above) is upregulated through sensing of HCV viral RNA in the cytoplasm. Therefore, increased levels of EGOT are detected shortly after infection even when UV inactivated non-replicative viruses are used (Carnero et al., 2016). Later in the infectious cycle, EGOT levels are increased in response to viral replication. Several sensor molecules are required for EGOT induction, including RIG-I and the non-canonical sensor PKR, which induce transcription through IRF3 and NF-κB, with the latter being required for EGOT transcription. Indeed, there is a good correlation between the levels of TNFα, which induces NF-κB, and cellular level of EGOT in liver tissues derived from HCV-infected patients, suggesting that TNFα could be a major driver of EGOT expression in the liver (Carnero et al., 2016).

As expected from a negative regulator of the IFN response (see above), EGOT depletion leads to decreased levels of viral genome and proteins and viral titers in HCV and SFV-infected cells (Carnero et al., 2016), likely due to increased levels of ISGs, such as GBP1, ISG15, Mx1, BST2, ISG56, IFI6 and IFITM1. Interestingly, some of the ISG targets of EGOT have been described as inhibitors of HCV or SFV entry, replication or release (Landis et al., 1998; Itsui et al., 2009; Raychoudhuri et al., 2011; Wilkins et al., 2013; Amet et al., 2014; Ooi et al., 2015). Kinetic experiments show that EGOT silencing leads first to increased levels of ISGs and then to decreased replication of HCV or SFV genomes. As discussed above, this repressive role of EGOT resembles what has been described for the lncRNAs NRIR and NRAV, negative regulators of the IFN pathway which are induced by IFN or infection (Kambara et al., 2014; Ouyang et al., 2014). Similar to what has been described for EGOT, downregulation of lncCMPK2/NRIR or NRAV lncRNA activates ISG transcription and inhibits HCV or influenza replication, respectively.

## Conclusion

*In vitro* studies on the role of lncRNAs in the IFN response in the absence of pathogens point to the presence of a strong perturbation in the expression of the non-coding transcriptome following IFN stimulation. From the very small number of studies on the role of lncRNAs in the antiviral response, it is evident that changes in the expression of this class of cellular effectors do play a critical role in negative regulation of the downstream steps of the IFN response. Together with proteins that act as negative regulators, this class of lncRNAs create a complex, decentralized regulatory network with overlapping and likely partially redundant functions which allows for extreme fine tuning of the magnitude and duration of the IFN response. However, in the presence of pathogens such as HCV, such negative feedback loops can favor viral replication and in some cases are actively hijacked by the virus to help in viral survival. There are two possible scenarios that can explain this from an evolutionary perspective. One possibility is that the co-evolution of the IFN response and pathogens has tipped in favor of viral survival due to the faster rate of viral evolution, with the viruses gaining the ability to exploit the immune response. Alternatively, it is plausible that at least in the case of some chronic infections, such a reduction in the magnitude of the immune response may be beneficial for the organism through limiting the damage incurred on the infected tissue. Future studies, by providing a more complete picture of the interplay of lncRNAs and viral infections, will shed additional light on the highly complex interplay of the antiviral response and pathogens.

## Author Contributions

SV and PF wrote and reviewed the manuscript. PF built the table and SV the figures.

## Conflict of Interest Statement

The authors declare that the research was conducted in the absence of any commercial or financial relationships that could be construed as a potential conflict of interest.
